# Differences in clinicopathological characteristics and computed tomography findings between signet ring cell carcinoma and nonsignet ring cell carcinoma in early and advanced gastric cancer

**DOI:** 10.1002/cam4.1417

**Published:** 2018-03-13

**Authors:** Jian Chen, Rong Cai, Gang Ren, Jianxi Zhao, Huali Li, Chen Guo, Wenguang He, Xiangru Wu, Wenjie Zhang

**Affiliations:** ^1^ Department of Radiology Xinhua Hospital Affiliated to Shanghai Jiaotong University School of Medicine Shanghai 200092 China; ^2^ Department of Radiotherapy Ruijin Hospital Affiliated to Shanghai Jiaotong University School of Medicine Shanghai 200025 China; ^3^ Department of Pathology Xinhua Hospital Affiliated to Shanghai Jiaotong University School of Medicine Shanghai 200092 China; ^4^ Department of Surgery Xinhua Hospital Affiliated to Shanghai Jiaotong University School of Medicine Shanghai 200092 China

**Keywords:** Comparative study, gastric cancer, multidetector computed tomography, signet ring cell carcinoma

## Abstract

Signet ring cell carcinoma (SRC) of the stomach is a histological type based on microscopic characteristics. SRC's clinicopathological characteristics and prognosis are still controversial. Our study is to describe the clinicopathological features and multidetector computed tomography (MDCT) findings of patients with SRC of the stomach in comparison with nonsignet ring cell adenocarcinoma (NSRC). We retrospectively analyzed data from 241 patients who had undergone curative gastrectomy, including 62 SRC and 179 NSRC. Clinicopathological outcomes and MDCT findings were evaluated, and we investigated whether these variables were correlated with histopathological type. In early gastric carcinoma, patients with SRC were younger (50.2 vs. 60.2 years; *P *=* *0.000) and more likely to be observed in the middle and lower third stomach (*P *=* *0.010). Early SRC had a tendency to be confined to the mucosa (82.1%). There were significant differences in degree of enhancement between early SRC and NSRC on MDCT imaging (*P* < 0.001). In advanced gastric carcinoma, SRC was more likely to be stage T3‐4 (100%). SRC patients had thicker tumors (*P* = 0.001) and a higher frequency of diffusely infiltrative gross appearance (*P* < 0.001). SRC was more likely to have high‐degree contrast enhancement than were NSRC (*P* = 0.001). The maximal diameter of SRC tumor on MDCT imaging correlated with lymph node metastasis (sensitivity 93.9%, specificity 74.1%) and serosal invasion (sensitivity 89.5%, specificity 78.0%) of SRC. In conclusion, SRC differs significantly from NSRC in clinicopathological features at presentation. MDCT could help differentiate advanced gastric SRC from NSRC based on the thickened stomach wall, high‐degree contrast enhancement, and a higher frequency of diffusely infiltrative gross appearance, particularly in combination.

## Introduction

Gastric cancer represents the second leading cause of cancer death and is the second most common invasive cancer in China. It is estimated that approximately 498,000 Chinese will die from gastric cancer in 2015 1. Gastric cancer is also a leading cause of cancer‐related deaths worldwide 2. Although the incidence of gastric cancer has declined in the last several decades, there is evidence of an increasing incidence of signet ring cell cancer (SRC) subtypes, comprising 11–37% of all gastric cancers in recent years 3, 4, 5. This increase in the proportion of SRC in cases of gastric adenocarcinoma can be explained by changes in the pathological classifications used to characterize these cancers 6.

SRC is a histological type of gastric carcinoma, primarily based on the microscopic characteristics of the tumor but not on the biological behavior as described by the World Health Organization 7. The WHO defines signet ring cell carcinoma as a poorly cohesive carcinoma composed predominantly of tumor cells with prominent cytoplasmic mucin and a crescent‐shaped nucleus eccentrically placed 8, 9. SRC carcinoma of the stomach is a histological type with controversial clinical outcomes, depending on whether it is early or advanced. According to several reports, advanced SRC of the stomach has worse outcomes than does NSRC 10, 11, 12, 13, 14, while early SRC has variously been reported to have a favorable outcome compared with that of NSRC 5, 10, 11, 15. It is clinically useful to be able to distinguish between SRC and NSRC in patients who have this cancer.

The role of computed tomography (CT) in the preoperative staging of gastric cancer, even if controversial, may be fundamental for evaluating the local extent and nodal involvement of the disease, especially in locally advanced cases. Currently, multidetector‐row computed tomography (MDCT) scanners allow thinner collimation and faster scanning, which markedly improves scanning resolution and enables rapid production of multiplanar reformation (MPR) images. Therefore, Shimizu et al. 16 reported that MDCT with the water‐filling method has advantages in terms of acceptable evaluation of depth invasion of gastric carcinomas and in visualization of histological changes in the tumors.

To the best of our knowledge, few studies have evaluated the effect of 64 MDCT for preoperative evaluation of SRC versus NSRC. We designed this study to retrospectively analyze the clinicopathological and MDCT features of SRC compared with other cell types in stomach cancer patients.

## Materials and Methods

### Patient selection

By performing a computerized search of pathology records, we selected 28 patients with early SRC and 34 patients with advanced SRC who had undergone MDCT examination preoperatively between 2002 and 2015 at Xinhua Hospital Affiliated Shanghai Jiaotong University School of Medicine, Shanghai, China. All patients underwent either total or partial gastrectomy, depending on the clinical stage of gastric cancer. The clinicopathological findings in patients with SRC and NSRC were also stratified according to early or advanced gastric carcinoma. For comparison with the SRC gastric cancer group, we randomly identified 179 patients with a diagnosis of NSRC (95 AGCs, 84 EGCs) who underwent surgery between the same periods. The time between contrast‐enhanced dynamic CT examinations and surgery was less than 1 week for all patients.

### Surgical approach

None of the patients included in this study received neoadjuvant treatment. Patients with localized adjacent peritoneal carcinomatosis underwent resection in a curative attempt. Patients with distant metastasis were generally contraindicated for surgery except in case of major symptoms such as gastric outlet obstruction, bleeding, or perforation. For patients undergoing resection with a curative intent, a total or distal gastrectomy was performed, depending on the location and macroscopic type of the tumor. Combined D2 lymphadenectomy was performed according to the rules of the third edition Japanese classification of gastric carcinoma 17. For local neoplasm of the cardia, a resection of the distal part of the esophagus followed by an esophagogastrostomy was included, whereas patients with located carcinomas of the antrum or pylorus underwent a classical subtotal (4/5) gastrectomy. For reconstruction, the Billroth II (after subtotal gastrectomy) or Roux–en‐Y (after total gastrectomy) techniques were performed.

### Histopathological evaluation

All of the tissues were examined by two independent experienced pathologists. Tumor tissue specimens were fixed with 10% buffered formalin and embedded in paraffin. Five‐micron‐thick sections through the largest tumor dimension were mounted on silanized slides, dewaxed with xylene, and dehydrated with ethanol. SRC was defined as >50% of the tumor consisting of isolated or small groups of malignant, cells containing intracytoplasmic mucin, according to the World Health Organization classification 7. Patients with a pathological diagnosis of SRC of the stomach were compared with the NSRC group. Patients were evaluated according to gender, age, tumor location, depth of tumor invasion, lymph node metastasis, distant metastases, TNM stage, peritoneal dissemination, lymphovascular invasion, and perineural invasion in accordance with the third edition Japanese classification of gastric carcinoma 18.

### CT technique

Computed tomography was performed with a 64‐channel MDCT (Somatom Definition, Siemens, Forchheim, Germany) or 256‐channel MDCT (Brilliance iCT256, Philips Medical Systems, Cleveland, OH). Each patient fasted for at least 6 h before undergoing CT. Each patient drank 750 mL water 1–2 h before the examination and drank 250 mL water again 15 min before the examination to distend the stomach. The CT parameters were as follows: detector collimation (1 mm), pitch (0.9), gantry rotation (0.5), tube voltage (120 kVp), tube current (240 mAs), matrix (512 × 512), slice thickness (5 mm), and reconstruction interval (1 mm). The arterial phase, portal venous phase, and equilibrium phase scan were obtained using a fixed 28 sec, 60 sec, and 120 sec equilibrium following intravenous injection of 100 mL of ionic contrast material at a rate of 3 mL/sec using an automatic injector with scan range from diaphragm to iliac crest. Coronal and sagittal section datasets were reconstructed with 3‐mm‐thick sections.

### Imaging analyses

Two board‐certified abdominal radiologists (R.G., C.J.) collectively and retrospectively reviewed the MDCT scans obtained in the 241 patients by consensus without knowledge of the pathological subtype of the gastric carcinoma.

The maximum diameter, tumor thickness, degree of enhancement, contrast enhancement pattern, macroscopic type in AGC, and high‐attenuating inner layer thickness in EGC were evaluated on CT scans. The Japanese classification of advanced gastric carcinoma was applied to adjust for the gross appearance of tumors. Gross appearance was classified as one of four types: type 1 are polypoid lesions, type 2 are ulcerating lesions surrounded by a thickened gastric wall with clear margins, type 3 are infiltrative ulcerating lesions, and type 4 are diffusely infiltrating lesions 18. The contrast enhancement patterns on parenchymal phase CT images were recorded as homogeneous, heterogeneous, or layered, which was defined as diffuse thickening of the gastric wall with more than 50% preservation of a multilayered pattern 19. The maximal diameter of tumor was the maximum diameter measured in the axial section of CT images and MPR images. The thickness of the tumor was measured at the thickest point of the most thickened wall, and the predominant thickened layer was recorded. For objective analysis, the CT attenuation value of the primary tumors on the axial CT image with the maximum diameter was measured, and the average CT value of any three measurements was selected. The ROI was drawn as large as possible to minimize noise, but care was taken to avoid partial volume effect. As the reflection of enhancement degree, ∆CT value = attenuation value (HU) in portal venous phase – attenuation value (HU) in unenhanced phase. The degree of enhancement the tumor was based on dynamic CT imaging using HU attenuation, where “low enhancement” if ∆CT was 6–20 Hu, “moderate enhancement” if ∆CT was 21–40 HU, and “high enhancement” if ∆CT was >40 HU.

### Statistical analysis

All statistical analyses were performed with SPSS for Windows version 20.0 (SPSS Inc, Chicago, IL, USA). Quantitative data were presented as the mean ± SD and evaluated with Student's *t*‐test or the Mann–Whitney *U*‐test. Qualitative data were evaluated with the χ^2^‐test or Fisher's exact test as appropriate. A *P*‐value of less than 0.05 was considered to be significant.

## Results

### Clinicopathological characteristics of patients according to histopathological types

Among the 241 enrolled patients, 150 were male (62%) and 91 were female (38%). The age range of patients was 15–88 years. The clinicopathological characteristics of 62 patients with SRC and 179 patients with NSRC are compared in Table 1.

**Table 1:** Comparison of the clinicopathological characteristics of patients with signet ring cell carcinoma (SRC) or nonsignet ring cell carcinoma (NSRC)

Characteristic	SRC (%)	NSRC (%)	*P* value
EGC (total, *n* = 112)	28	84	
Gender			
Male	12 (42.9)	51 (60.7)	0.099
Female	16 (57.1)	33 (39.3)	
Age (years, mean ± SD)	50.18 ± 1.918	60.15 ± 1.248	0.000
Tumor location			
Upper third	0 (0.0)	12 (14.3)	0.010
Middle third	10 (35.7)	16 (19.0)	
Lower third	18 (64.3)	56 (66.7)	
Depth of tumor invasion			
Mucosa (T1a)	23 (82.1)	43 (51.2)	0.004
Submucosa (T1b)	5 (17.9)	41 (48.8)	
LN metastasis			
Negative	25 (89.3)	68 (81.0)	0.394
Positive	3 (10.7)	16 (19.0)	
Removed lymph nodes, mean ± SD	0.25 ± 0.160	0.86 ± 0.370	0.316
Macroscopic finding			
Elevated	2 (7.1)	12 (14.6)	0.430
Flat	4 (14.3)	7 (8.5)	
Depressed	22 (78.6)	62 (76.8)	
AGC (total, *n* = 129)	34	95	
Gender			
Male	21 (61.8)	66 (69.5)	0.410
Female	13 (38.2)	29 (30.5)	
Age(years, mean ± SD)	57.65 ± 2.127	65.26 ± 1.127	0.002
Tumor location			
Upper third	5 (14.7)	25 (26.3)	0.490
Middle third	9 (26.5)	24 (25.3)	
Lower third	19 (55.9)	42 (44.2)	
Entire	1 (2.9)	4 (4.2)	
LN metastasis			
Negative	2 (5.9)	19 (20.0)	0.056
Positive	32 (94.1)	76 (80.0)	
pT stage			
pT2	0 (0.0)	14 (14.7)	0.009
pT3	13 (38.2)	35 (36.8)	
pT4	21 (61.8)	46 (48.4)	
pN stage			
pN0	2 (5.9)	21 (22.1)	0.099
pN1	9 (26.5)	17 (17.9)	
pN2	5 (14.7)	20 (21.1)	
pN3	18 (52.9)	37 (38.9)	
pM stage			
pM0	31 (91.2)	83 (87.4)	0.758
pM1	3 (8.8)	12 (12.6)	
pTNM stage			
1	0 (0.0)	6 (6.3)	0.158
2	8 (23.5)	27 (28.4)	
3	23 (67.6)	50 (52.6)	
4	3 (8.8)	12 (12.6)	
Peritoneal dissemination			
Positive	3 (8.8)	6 (6.3)	0.697
Negative	31 (91.2)	89 (93.7)	
Lymphovascular invasion			
Absent	26 (76.4)	70 (73.6)	0.292
Present	4 (11.8)	20 (21.1)	
Unknown	4 (11.8)	5 (5.3)	
Perineural invasion			
Absent	26 (75.5)	76 (80.0)	0.664
Present	8 (23.5)	19 (20.0)	

The clinicopathological characteristics of early SRC were compared with those of NSRC, and significant differences were observed with respect to age, tumor location, and depth of tumor invasion. Patients with early SRC tended to be younger (50.18 vs. 60.15 years, *P* = 0.000). Early signet ring cell carcinoma was more likely to be observed in the middle and lower third stomach (*P* = 0.010). SRC had a larger proportion of mucosa‐confinement than did NSRC among early gastric carcinoma patients (82.1% vs. 51.2%, *P* = 0.004). The proportion of female patients in the group with SRC was larger than in NSRC without a statistically significant difference (57.1% vs. 39.3%, *P* = 0.09). There were no differences in gender, lymph node metastasis, number of involved lymph nodes, or macroscopic type between patients with early SRC and those with early NSRC.

Among the patients who underwent gastrectomy of advanced gastric carcinoma, SRC was again more commonly observed in younger patients (57.65 vs. 65.26 years, *P* = 0.002). pT3 and pT4 carcinomas were observed more frequently in patients with SRC than in those with NSRC (38.2% vs. 36.8%; 61.8% vs. 48.4%; *P* = 0.009). The SRC group showed a higher lymph node metastasis rate in contrast with the NSRC group, but there was no significant difference (94.1% vs. 80.0%, *P* = 0.056). There were no significant differences in tumor location, pN stage, pM stage, pTNM stage, peritoneal dissemination, lymphovascular invasion, or perineural invasion between SRC and NSRC in advanced gastric carcinoma (Table 1).

### CT features

**Figure 1:**
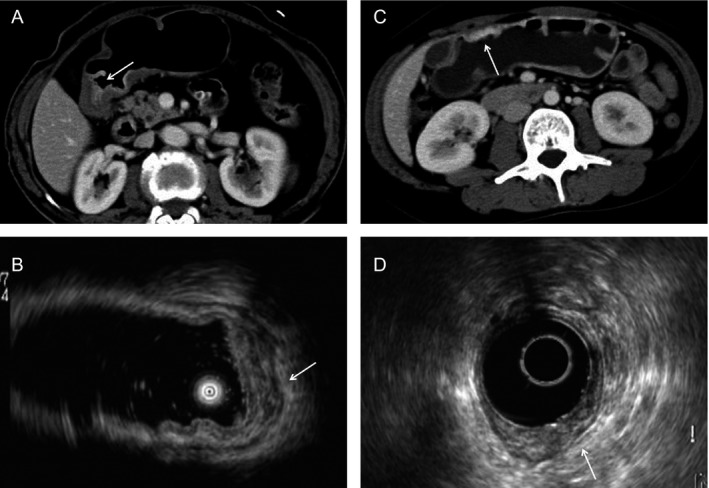
Contrast‐enhanced computed tomography images and corresponding endoscopic ultrasonography (EUS) images of early gastric carcinoma. (A, B) 72‐year‐old woman with early SRC. Contrast‐enhanced CT scan obtained during the parenchymal phase shows focal inner wall thickening (arrow). EUS image of the lesion shows an echo‐poor, inhomogeneous lesion. Surgical resection confirmed signet ring cell carcinoma infiltrated to the mucous layer. (C, D) Elevated early NSRC in a 38‐year‐old woman. The attenuation of the enhancing thickened gastric wall is higher than that of the SRC (arrow). EUS image of the lesion shows a hypoechoic lesion spreading from the mucosal to submucous layers.

The MDCT imaging features of EGC with SRC and NSRC are summarized in Table 2. The contrast enhancement degrees of the two types of carcinoma were significantly different (*P* = 0.000, Fig. 1). A higher proportion of low degree of enhancement (42.9%) was observed in SRC. Differences in tumor thickness were not statistically significant (*P* = 0.906). There were no significant differences between groups in terms of maximal diameter of tumor and thickness of high‐attenuating inner layer.

**Table 2:** Comparison of multidetector‐row computed tomography (MDCT) features of patients with SRC and NSRC in early gastric carcinoma

MDCT features	SRC (%) (*n* = 28)	NSRC (%) (*n* = 84)	*P*
Maximal diameter of tumor (cm), mean ± SD	2.56 ± 0.20	2.46 ± 0.11	0.474
Thickness of tumor (cm), mean ± SD	1.02 ± 0.07	1.05 ± 0.04	0.906
Thickness of high‐attenuating inner layer (cm), mean ± SD	0.45 ± 0.63	0.44 ± 0.22	0.546
Degree of enhancement			
High	11 (39.3)	40 (47.6)	0.000
Moderate	5 (17.9)	36 (42.9)	
Low	12 (42.9)	8 (9.5)	

**Figure 2:**
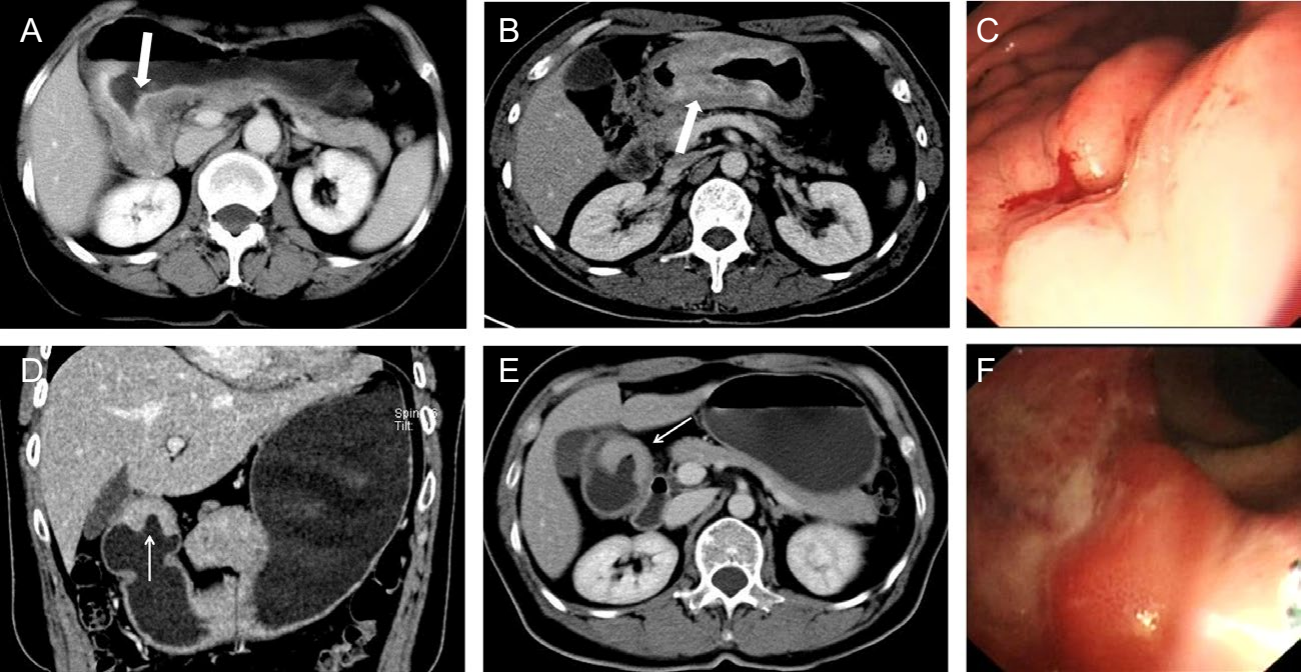
Contrast‐enhanced computed tomography images of advanced gastric carcinoma. (A, B) Two contrast‐enhanced CT images in different patients with advanced SRC. Contrast‐enhanced CT scan shows diffuse gastric wall thickening with strongly enhancement. The layered and heterogeneous‐enhancement pattern is shown. (C) Endoscopic image (same patient in B) of the lesion shows a diffusely infiltrating lesion. (D–F) 55‐year‐old man with NSRC. Contrast‐enhanced CT scan and coronal reconstruction show focal gastric wall thickening mainly of the enhancing thickened inner layer (arrow). The homogeneous‐enhancement pattern is shown. Endoscopic image of the lesion shows an ulcer lesion located in the gastric antrum.

The MDCT imaging features of AGC with SRC are summarized in Table 3. Among the patients with AGC, on the axial CT images, all SRC manifested focal or diffuse wall thickening. The tumor thickness was significantly greater in SRC than in NSRC (1.72 cm vs. 1.49 cm, *P* = 0.001). SRC tumors exhibited a more substantial enhancement (*P* = 0.001) (Fig. 2). The most common contrast enhancement pattern in SRC and NSRC was the homogeneous type. A difference in the macroscopic type between SRC and NSRC was observed. The proportion of type 1 and type 4 in patients with advanced SRC was significantly larger than in those with NSRC (17.6% vs. 3.2%, 41.2% vs. 13.7%, *P* = 0.000). A layered type was seen more often in SRC (six [17.6%] patients) than in NSRC (five [5.3%] patients), although there was no significant difference (*P* = 0.101). No significant differences were observed between the SRC and NSRC patients regarding maximal diameter of tumor (Table 3). In addition, only one patient with SRC showed miliary punctate calcifications in the diffusely infiltrative lesions.

**Table 3:** Comparison of multidetector‐row computed tomography (MDCT) features of patients with SRC or NSRC in advanced gastric carcinoma

MDCT features	SRC (%) (*n* = 34)	NSRC(%) (*n* = 95)	*P*
Maximal diameter of tumor (cm, mean ± SD)	5.86 ± 0.41	5.10 ± 0.21	0.102
Thickness of tumor (cm, mean ± SD)	1.72 ± 0.64	1.49 ± 0.53	0.001
Degree of enhancement			
High	26 (76.5)	40 (42.1)	0.001
Moderate	6 (17.6)	50 (52.6)	
Low	2 (5.9)	5 (5.3)	
Enhancement pattern			
Layered type	6 (17.6)	5 (5.3)	0.101
Heterogeneous type	13 (38.2)	37 (38.9)	
Homogeneous type	15 (44.1)	53 (55.8)	
Macroscopic type			
Type 1	6 (17.6)	3 (3.2)	0.000
Type 2	11 (32.4)	66 (69.5)	
Type 3	3 (8.8)	13 (13.7)	
Type 4	14 (41.2)	13 (13.7)	

### Relationship between CT features and clinicopathological characteristics in SRC

Univariate analysis was performed on the relationship between clinicopathological characteristics and CT features in patients with SRC (Table 4). There were 27 cases of perigastric lymph node metastasis confirmed in patients with SRC, and the remaining 35 cases showed no metastasis. The tumor maximal diameter and thickness of metastasis patients was significantly higher than those of the nonmetastasis patients (5.71 ± 0.42 vs. 2.63 ± 0.21 cm; 1.66 ± 0.72 vs. 1.06 ± 0.07 cm, respectively; *P* = 0.000). Serosal invasion or adjacent structure invasion (T4 carcinomas) was observed in 21 patients with SRC. The tumor maximal diameter, thickness, and enhancement degree were all closely related to depth of tumor invasion (*P* < 0.001).

**Table 4:** Relationship between the MDCT features and clinicopathological parameters in SRC

Clinicopathological characteristics	MDCT features						
	No. of patients, *n*	Maximal diameter of tumor (cm) mean ± SD	*P*	Thickness of tumor (cm), mean ± SD	*P*	Enhancement degree (HU)	*P*
LN metastasis							
Negative	27	2.63 ± 0.21	0.000	1.06 ± 0.07	0.000	35.0 ± 0.47	0.22
Positive	35	5.71 ± 0.42		1.66 ± 0.72		48.91 ± 4.10	
Depth of tumor invasion							
T1–T3	41	3.28 ± 2.24	0.000	1.20 ± 0.07	0.000	35.98 ± 2.89	0.001
T4	21	6.50 ± 0.59		1.79 ± 0.08		56.29 ± 5.32	

**Figure 3:**
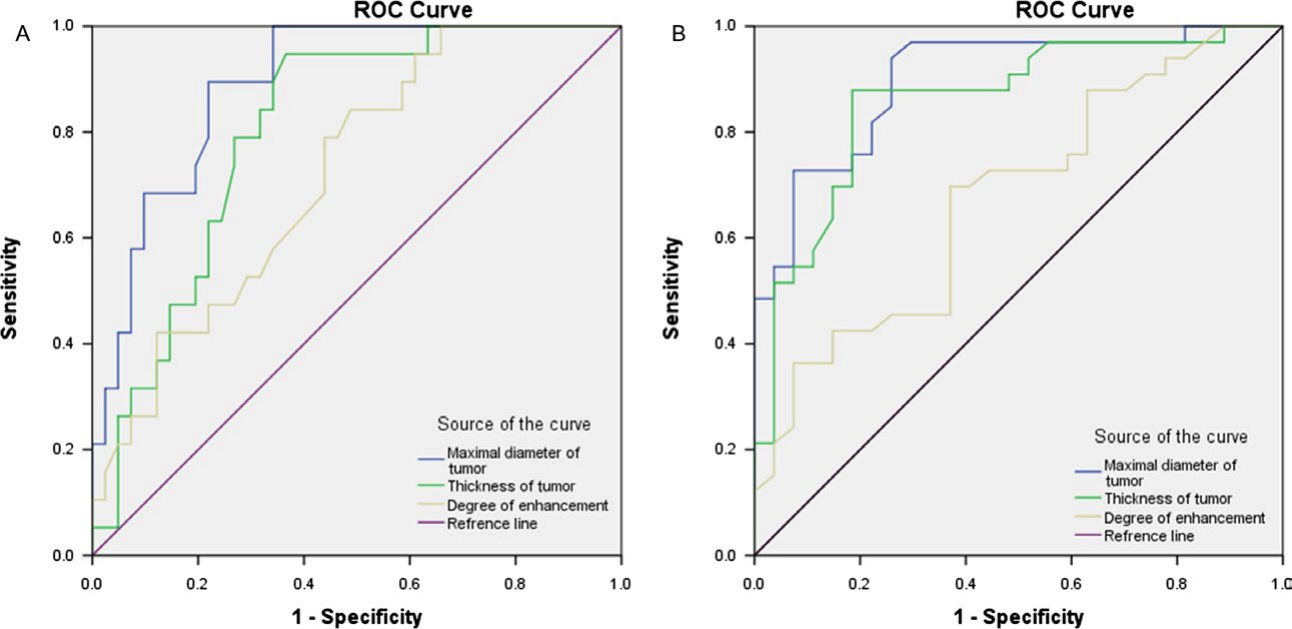
Performance of the MDCT characteristics for the diagnosis of the serosal invasion and lymph nodes metastatic of SRC tumors. (A) Receiver operating characteristic curves (ROCs) for the CT characteristics in determining the serosal invasion of SRC. The areas under the ROC curves (AUCs) for the maximal diameter, thickness and degree of enhancement were 0.89, 0.80 and 0.72. (B) ROCs for the CT characteristics in differentiating metastatic and non‐metastatic lymph nodes. AUCs for the maximal diameter, thickness and degree of enhancement were 0.90, 0.86 and 0.68.

Receiver operating characteristic (ROC) analysis showed that the accuracy of the maximal diameter of tumor in determining the lymph node metastasis and serosal invasion of SRC tumors was relatively high (areas under ROC curve [AUC] were 0.901 and 0.890, respectively) (Fig. 3). Meanwhile, 3.12 cm and 4.29 cm were considered to be the best predictive cutoff values for lymph node metastasis (sensitivity 93.9%, specificity 74.1%) and serosal invasion (sensitivity 89.5%, specificity 78.0%), respectively. In addition, AUCs of the thickness of tumor and the degree of enhancement in differentiating metastatic and non‐metastatic lymph nodes were 0.859 and 0.676, respectively. In determining the serosal invasion of SRC tumors, AUCs of the thickness of tumor and the degree of enhancement were 0.803 and 0.720, respectively.

## Discussion

SRC of the stomach is thought to arise in gastric mucosa that is not metaplastic and is confined to the glandular neck region in the proliferative zone 20. SRC is a subtype of gastric carcinoma that is distinct from other gastric adenocarcinomas at presentation. Studies have shown that subtypes of gastric cancer with histological distinctions can be distinguished by gene expression data, which suggest that SRC may be a completely distinct entity 3.

Although there are already many survival studies comparing gastric carcinoma with SRC and NSRC, SRC has a highly controversial prognosis. According to previous studies, the prognosis of signet ring cell adenocarcinoma is debated and appears to depend on the stage of the cancer. Most studies of early gastric cancer have reported that early SRC displays favorable behavior compared with NSRC types. Woo et al. 21 and Jiang et al. 11 have reported that the survival rate of SRC‐type EGC was significantly better than that of other types. Conversely, in advanced gastric cancer, the prognosis of SRC is more controversial and is commonly thought to be poor. Retrospective studies from China with advanced SRC showed a significantly worse five‐year survival rate than with NSRC 14, 22. Another study showed that SRC in stage III has the highest risk of death after stratifying stages when compared to well or moderately differentiated (WMD) and poorly differentiated (PD) tumors 5. Thus, it is clinically useful to differentiate gastric cancer with SRC types from other types of gastric carcinoma, whether in EGC or in AGC.

According to several studies, the clinicopathological features of early SRC are notably different from NSRC, in terms of the depth of invasion and lymph node metastasis 15, 21. Other groups have reported that they determined no significant differences between early SRC and NSRC with regard to lymph node metastasis 11, 23. Our findings indicate that early SRC has a distinct presentation when compared to NSRC. Early SRC had a larger proportion of mucosa‐confined lesions (*P* = 0.004). Whereas NSRC presents more proximally, SRC was more likely to present in the middle or lower stomach (*P* = 0.010). Early SRC was observed in younger patients (*P* = 0.000) and in female patients compared with NSRC. These results are consistent with previous studies 2, 15. The reason that SRCs are predominant in younger and female patients remains unclear. There is a theory that histology may be influenced by sex hormones 24, 25. It is suggested that better overall survival of early SRC identified in most studies may be related to the younger age and more frequently mucosa‐confined lesions in SRC patients 6. Additionally, lymph node metastasis, which is important prognostic factor, has not shown significant differences between early SRC and NSRC in the present study, similar to previous studies 11, 26.

In advanced gastric cancer, according to previous studies, signet ring cell carcinoma appears to present at later stages, with a greater proportion of patients presenting at stage 4 with more advanced TNM stage, and higher tumor grade 3, 13. We also observed among patients with AGC that SRC was more associated with a T3/T4 tumor (100% vs. 85.2%, *P* < 0.05) and N3 tumor (52.9% vs. 38.9%), which may indicate that SRC was associated with more aggressive tumors. Consistent with previous studies 14, 22, higher LN metastasis rates were observed in advanced SRC, even though there was no statistical significance (*P* = 0.056). It is worth noting that the gross appearances of SRC and NSRC were different (*P* = 0.000). The most common appearance was type 4 in SRC and type 2 in NSRC. Taghavi et al. 3 reported that signet ring cell carcinoma is more likely to present with an overlapping location. This phenomenon was also observed in our study.

The ability to distinguish SRC from NSRC on the basis of CT imaging characteristics of these tumors is clinically important because CT is the imaging technique that is most frequently used for the detection and preoperative staging of gastric cancer. By combining the helical scanning technique with this water‐filling method, the accuracy of the depiction of gastric cancer and its depth of invasion by CT has remarkably improved. High‐quality MPR images allow visualization of the fine anatomic details of the gastric wall in any plane. The accuracy of 64‐slice MDCT in determining tumor penetration depth was superior to that obtained by single‐detector and 4‐slice MDCT 27, 28. In the present study, the degree of contrast enhancement was lower in EGC with SRC than with NSRC. This finding needs to be further studied. Other than EGC, there were more significant differences between advanced SRC and NSRC on MDCT. Most of advanced SRC showed a thickening of the diffusely infiltrating lesions in our study. The result was consistent with the previous reports of clinicopathological characteristics in SRC, showing that Borrmann type III and type IV were the most common patterns in AGC with SRC 11, 14. This finding may arise from the ability of SRC to diffusely infiltrate the gastric wall and cause a marked scirrhous reaction that is observed pathologically. Also, compared with NSRC, the AGC with SRC patients were observed to have a greater incidence of high‐degree contrast enhancement (*P* = 0.001). This phenomenon was also observed in the study by Lee et al. 29. There is a theory that the degree of contrast enhancement on dynamic CT scans was correlated with vascular endothelial growth factor (VEGF) expression and microvessel density (MVD) 30. Therefore, we supposed that SRC inducing more neovascularity than NSRC may lead to the high degree of enhancement on CT scans. Li et al. 14 reported that mean tumor size tended to be larger in SRC than in NSRC, although other studies 15, 31 showed that mean tumor size was smaller in SRC. In the present study, advanced SRC patients were observed to have larger (*P* = 0.102) and thicker (*P* = 0.001) tumors on the MDCT images. Although both mucinous adenocarcinoma and SRC are mucin‐secreting adenocarcinomas, the typical layered enhancement pattern in mucinous adenocarcinoma was not observed in advanced SRC.

Lymph nodes metastasis and serosal invasion was reported to be two well‐known prognostic factors for survival of gastric carcinoma. Yokota et al. 32 showed that tumor size was not an independent prognostic factor in patients with gastric carcinoma. In contrast, another study reported that tumor size clinically served as a simple predictor of tumor progression and survival in patients with gastric carcinoma 33. In our study, the accuracy of the maximal diameter of tumor on MDCT in determining lymph node metastasis and serosal invasion of SRC tumors was higher than that of the enhancement degree and thickness of SRC tumors. A previous study reported that, in the absence of lymphatic invasion, mucosal SRC with tumor sizes <15 mm had no lymph node metastasis 34.

There were several limitations in this study. First, the retrospective nature of this study may introduce some bias. Second, we evaluated only those CT images during the portal phase, limiting our ability to analyze dynamic changes in the enhancement pattern. Third, the ROI was obtained to minimize noise and to avoid partial volume effects. Thus, the current analysis has a potential sampling error. Although a pixel‐by‐pixel analysis would have been desirable, it would have required the use of a special computer program that was not available. Moreover, because our observational period was relatively short, we could not analyze the clinical outcomes based on the parameters obtained by MDCT scans and histopathological surveys. A further follow‐up study with a larger number of patients may be required to confirm our results.

In conclusion, SRC is a distinct type of gastric carcinoma in terms of clinicopathological characteristics and CT features. We observed statistically significant differences in MDCT findings between the patients with advanced SRC and those with NSRC. A diffusely thickened and high degree of enhancement can be helpful for findings that suggest signet ring cell carcinoma of the stomach. The maximal diameter of tumor on MDCT scans is useful in determining not only the serosal invasion of gastric SRC but also the extent of lymph node metastasis.

## Conflicts of Interest

The authors have disclosed that they have no significant relationships with, or financial interest in any commercial companies pertaining to this article.
